# Finite Element Modelling of a Reflection Differential Split-D Eddy Current Probe Scanning Surface Notches

**DOI:** 10.1007/s10921-020-00673-6

**Published:** 2020-03-17

**Authors:** Ehsan Mohseni, Demartonne Ramos França, Martin Viens, Wen Fang Xie, Baoguang Xu

**Affiliations:** 1 Department of Electronics & Electrical Engineering, Centre for Ultrasonic Engineering (CUE), University of Strathclyde, 99 George Street, Glasgow, G1 1RD UK; 2 John Abbott College, 21275 Lakeshore Dr, Sainte-Anne-de-Bellevue, Québec H9X 3L9 Canada; 3 Département de génie mécanique, L’École de Technologie supérieure, 1100 Rue Notre-Dame O, Montreal, Québec H3C 1 K3 Canada; 4 Department of Mechanical & Industrial Engineering, Concordia University, 1455 De Maisonneuve Blvd. W., Montreal, Québec H3G 1M8 Canada

**Keywords:** Eddy current testing, Split-D reflection differential probe, Absolute probe, Finite element analysis

## Abstract

Differential eddy current probes are commonly used to detect shallow surface cracks in conductive materials. In recent years, a growing number of research works on their numerical modelling was conducted since the development of analytical or semi-analytical models for such a sensor may be prone to intractable complications. In this paper finite element modelling (FEM) has been employed to simulate the interaction of a reflection differential split-D probe with surface electrical discharge machined (EDM) notches in 3-dimensional (3-D) half-space. In order to attain a better insight into the correct setup of the FEM parameters, a simple multi-turn cylindrical absolute coil has also been modelled. The outcome generated through the simulated scan of this absolute coil over a surface notch in aluminum is validated with existing experimental impedance data taken from the literature. Parameters contributing to reliable FEM simulation results, such as maximum mesh size, mesh distribution, the extent of the surrounding air domain and conductivity of the air are investigated for the 3-D modelling of both absolute and differential probes. This study shows that the simulation results on a commercial reflection differential split-D surface pencil probe closely estimate the experimental measurements of the probe’s impedance variations as it scans three EDM notches having different depths in aluminum. The simulation results, generated by Comsol Multiphysics FEM package (COMSOL I, COMSOL multiphysics reference manual, version 5.3, COMSOL AB, 2018, www.comsol.com), for the cases of absolute and differential probes are checked for their extent of validity.

## Introduction

The materials’ structure in manufactured parts is almost never free of microscopic imperfections. Under specific loading and environmental conditions, these imperfections may grow and form critical discontinuities. For instance, the fatigue crack is a very common defect type that could be frequently found in components under cyclic loads. These defects may deleteriously affect the performance of components and industrial systems by reducing their expected lifetime. In-service non-destructive testing (NDT) of components and systems is crucial when these systems are directly related to human safety. As an instance, aerospace industry, among all the industries that employ NDT to assess the integrity of structures [1–4], is tightly connected to human safety. A wide variety of fatigue-induced flaws may exist in the aircraft components, which requires having periodical inspections in place [5, 6]. Depending on the nature and location of these flaws, proper NDT techniques should be assigned for in-service inspections. Eddy current testing (ECT) has been routinely employed in various industries as a well-established NDT technique applied to conductive materials. Recent signs of progress in ECT is readily found in thickness measurement of coatings or thin conductive sheet metals, evaluation of conductivity and permeability variations, detection of surface and near-surface breakings such as cracks [7–9]. This wide diversity of applications has demanded the development of different configurations for eddy current (EC) sensors tailored to specific inspection purposes [10]. In the aerospace industry, ECT is well-known for its superior inspection performance of bolt holes, lap joints, wheels and engine components [11–13].

Absolute surface probes, with their simplest configuration (a single multi-turn circular coil), are typically conceived to operate at a frequency range of 100 Hz to 4 MHz. They may possess different shapes and sizes depending on the type of application. Low-frequency absolute surface probes (no more than 500 kHz) with relatively large footprint diameters are best suited to either evaluate thickness, permeability and conductivity of the components or detect near-surface defects and inhomogeneities since their lower operating frequencies imply higher penetration depths inside the material [9]. Reflection differential and bridge differential probes have more elaborated designs. These probe types are frequently employed to detect short and shallow surface breakings. Besides being virtually insensitive to gradual variations in the material’s thickness, conductivity, and permeability, the reflection differential probes compensate for the unwanted effects of the probe’s tilt or lift-off. Surface differential probes are commonly used in the form of an optimized reflection differential split-D configuration, which has a relatively small footprint because of the shape and stacking of its receiver coils.

Nowadays, the advanced electronics and computing systems facilitate the use of discretization approaches such as finite element method (FEM) on a regular basis in industries and universities. Although FEM may not be the best in terms of solving speed, several well-established commercial FEM software packages are currently conceived for a wide variety of problem classes with appropriate governing equations and solvers. In the present paper, Comsol is extensively used to model the interaction between eddy currents and geometrical discontinuities in aluminum.

Considering the advantages associated with the surface differential probes, a growing number of researchers focused on the modelling of this type of configuration. Most of the current works are devoted to numerical modelling and parametric studies of split-D differential probes since the experimental tests may be time-consuming and costly [14–21]. For instance, in the work developed by Mooers et al. [15, 16], the results obtained from two numerical software packages, namely VIC-3D and EC SIM, were compared with experimental measurements of a split-D probe. VIC-3D and EC SIM were employed again to conduct a parametric sweep on the dimensions of each constituting component of a split-D probe, and the influence of each parameter on the recorded signals was studied as the probe scanned a notch [17]. A model developed by Nakagawa et al. [19] for a split-D probe described the effect of electrical discharge machined (EDM) notch width on the probe’s output signal, and simulation results were then validated with experimental tests. The primary objective in most of these modelling approaches is to reproduce the experimental results to the highest possible accuracy since a valid EC model can form the foundation of defect characterization (inversion), and reliability studies [22–25]. Therefore, it is of high importance to conduct a proper validation study to assess the rigorousness of each EC model [14, 18, 26]. Rosell and Persson [20] reproduced the results of a numerical benchmark study called TEAM problem 8, which was established by the TEAM workshop [27]. They modelled a differential probe comprised of one driver and two circular receiver coils using Comsol. Despite their interesting results, the probe’s dimensions were significantly larger than those of commercially available split-D probes, and the frequency investigated is much lower than the typical operating frequency of these probe types. Certainly, all those ideal considerations are key for providing a basis to validate a wide range of modelling efforts. However, they are not sufficiently robust for validating the models targeting the inspection of shallow fatigue cracks.

Unlike the previous studies where proprietary software and codes were employed to model the interaction of split-D probes with surface defects, the present study uses Comsol Multiphysics as a flexible and accessible modelling tool to optimize the model parameters for two different probes; a surface absolute coil and a split-D reflection probe, scanning over surface notches. In the first stage of this paper, a model for an absolute surface coil is prepared according to Burke’s benchmark study in order to refine the modelling parameters using a simpler probe geometry [28]. Afterward, the realistic geometry of a split-D probe is modelled, and the inspection results are compared with measurements. To this end, the experimental setup used to validate numerical model is described in Sect. 2. It comprises a commercially available split-D differential probe scanning three notches with different depths in an aluminum calibration block. Then, in Sect. 3, the model for the absolute probe is presented and the results are compared against the reference values found in the literature [28]. The commercial split-D probe is modelled in Comsol in Sect. 4 where numerical simulations are carried out for all three reference notches in the aluminum calibration block and compared to the measurement data. Finally, the summary and conclusions are given in Sect. 5. The validated model for the split-D probe will be used to conduct a model-assisted probability of detection study and to provide training data sets for a fuzzy logic-based inversion algorithm [29]. For these very purposes, the validity of Comsol in the estimation of the split-D probe’s output signals as it scans surface EDM notches is investigated systematically.

## Impedance Measurement of a Commercial Split-D Coil

**Fig. 1:**
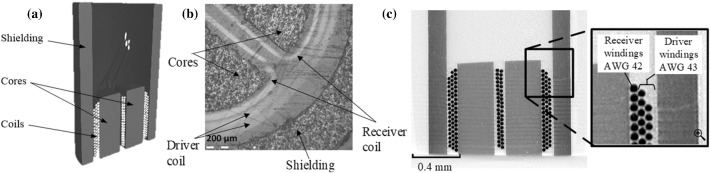
**a** 3-D model of the split-D probe showing coils, cores and magnetic shielding. **b** Zoom on the probe’s tip taken with a confocal optical microscope showing the outer driver coil and the inner D-shaped receiver coils. **c** A 2-D section of the probe’s 3-D model generated by X-ray CT scan showing the windings of the driver and receiver coils

In order to verify Comsol Multiphysics potential in simulating the output response of a commercial split-D probe, a Nortec-500 flaw detector from Olympus NDT corporation along with a reflection differential split-D surface pencil probe are selected. The probe’s bandwidth extends from 500 kHz to 3 MHz. The probe is connected to Nortec, and the probe’s impedance is measured as it scans three distinct EDM reference notches engraved in an aluminum calibration block. Figure 1a and b give the details on the probe’s tip, showing the driver coil, receiver coils inside the driver, surrounding ferrite shielding and D-shaped ferrite cores. Some features related to the probe’s configuration and geometry such as number of turns for driver and receiver coils, the wire gauge for the coils, and the relative magnetic permeability of the ferrite cores and shielding were provided by manufacturer. The variations in the magnetic permeability of the ferrite cores due to the changing electromagnetic field of the probe is neglected, as the simulations suggest that the magnetic flux density within the cores is extremely small. the Measurements on the probe’s 3-D model, reconstructed by a Nikon XTH 225 X-ray micro CT scan, and on the microscopic images are carried out to indicate the remaining dimensions of the probe constituents. Figure 1c shows a 2-D section of the 3-D model reconstructed from X-ray images for the probe’s geometry. Table 1 summarizes all relevant information on the dimensions and material properties of the probe’s constituents.

**Table 1:** Dimensions and material properties of the commercial split-D probe’s components

Receiver D-coils:	20 loops of 0.063 mm dia. wire	Shielding inner diameter:	1.934 mm
Driver coil:	37 loops of 0.055 mm dia. wire	Shielding outer diameter:	2.528 mm
Coils height:	1.260 mm	Shielding height:	3.000 mm
Core:	1.254 mm dia. × 2.000 mm height	Conductivity of cores and shielding:	0.2 S/m
Gap between cores:	0.224 mm	Permeability of cores and shielding:	2500 µ_0_

The Nortec-500 flaw detector has a built-in screen to display the probe’s differential impedance. However, the impedance measurements shown on the complex impedance plane of Nortec can only be recorded in terms of voltages through Nortec’s analog output channels. For recording the data from these channels, a LabVIEW application together with a data acquisition card are used. Therefore, the voltage signals, respectively given by the horizontal and the vertical position of the probe impedance on the detector screen, are used to verify the accuracy of shape, phase, and amplitude of the simulated signals in Comsol.

A 95 mm long × 35 mm wide × 5 mm high calibration 7075-T6 aluminum block containing three 0.18 mm-wide EDM notches with different depths (namely, 0.188 mm, 0.503 mm and 1.008 mm) is used throughout the scan measurements. These notches extend throughout the width of the aluminum block, so their lengths correspond to 35 mm implying they are much longer than the probe’s footprint. The conductivity of this aluminum block is measured as 19.7 MS/m using the Nortec 500 flaw detector itself and a dedicated conductivity probe.

**Fig. 2:**
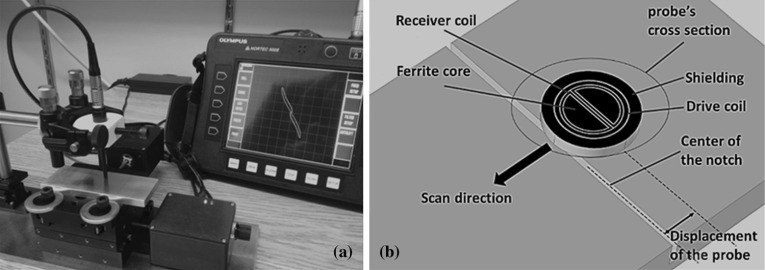
**a** Experimental setup for measuring the split-D probe’s differential impedance as the probe scans three reference notches with different depths in aluminum. **b** Schematic of the split-D probe’s orientation and scan direction with respect to an EDM notch

For the experimental setup, the split-D probe is clamped inside an alignment device. The aluminum block sits on a motorized X–Y table allowing micrometric translations along each axis. Initially, the horizontal and vertical gains of the Nortec-500 flaw detector are equally adjusted, and a 6 V-driving voltage is applied to the probe. With Nortec, it is important to keep the same impedance plane rotation angle during all scans in order to be able to draw comparisons between simulation results and experimental measurements. In this case, the angle of zero degrees is selected. In order to introduce an initial lift-off to the probe, the probe’s tip is placed on the aluminum block, and then the probe’s lift-off is varied using a micrometric Z-stage in a manner to have a small lift-off of 30 ± 10 µm. It is notable that the probe’s lift-off is only set once at the scan’s start point and the error in lift-off adjustment is dictated the limited precision of the micrometric knob on the Z-stage. Each notch is scanned five times and each time, the lift-off is reset to 30 ± 10 µm in order to include the lift-off adjustment errors. This procedure is essential since the lift-off calibration can vary slightly from one scan to another. In addition, the rotation of the probe is finely adjusted in order to set the separation surface of the D-cores parallel to the notch axis. Although it is known that achieving a perfect alignment for the probe’s rotation is not possible, but according to a study on angular variations of a split-D probe by Mooers and Aldrin, small angular rotations of the probe between − 10 and 10 degrees does not affect either shape or the amplitude of the probe’s signal [30]. The perpendicularity of the probe with respect to the aluminum block’s surface has been verified by scanning a surface notch. The system’s alignment was confirmed by a fairly symmetric 8-shaped signal with positive and negative peaks of almost equal magnitudes. Notches are scanned by moving the center of the probe's tip from − 2 to + 2 mm with respect to the notch centerline. The data is recorded in steps of 50 µm along the scanning path. Figure 2a and b illustrate the experimental setup and the probe’s scan direction with respect to a single notch, respectively.

## Refinement of Finite Element Modelling Parameters Using an Absolute Coil

The optimized EC system modelling parameters in Comsol are discussed in this section so as to reproduce experimental data provided in Burke's experiment [28]. Burke presented the impedance variation measurements of an air-cored absolute coil scanning over a rectangular notch in an aluminum plate. The detailed description of the setup, geometries, and dimensions can be found in the original work [28].

**Fig. 3:**
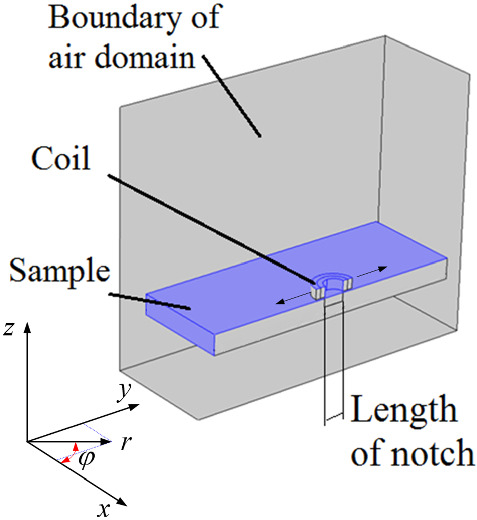
The half 3-D model developed in Comsol to reproduce the results presented in [34] for an absolute coil EC probe. A circular coil, a defective aluminum plate, and the encompassing air domain are shown

The axisymmetric nature of an eddy current absolute coil’s geometry allows one to build a two dimensional (2-D)-axisymmetric model when the coil is either in air or located over an un-defective conductor. However, the geometry is no longer axisymmetric as soon as a notch introduced onto the conductor’s surface. Consequently, Burke's experimental arrangement needs to be modelled in 3-D. Nonetheless, because the coil is always centered on the notch axis, the problem is symmetric with respect to a plane perpendicular to the conductor's surface and passing through the notch’s centerline. The full-scale geometry of the Burke’s setup model could thus be cut in half using this plane of symmetry. The overall half 3-D model created in Comsol is illustrated in Fig. 3.

The model comprises an aluminum block with a rectangular surface notch located in the middle of the block’s surface, a hollow cylindrical conductor representing a coil and a rectangular prism domain encompassing all the components. All the dimensions used in this model are taken from [28], except for the sizes of the aluminum block and the air domain, which are set to minimize the runtime of the finite element (FE) solver. The material properties of aluminum, as taken from [28], is assigned to the defective block. The properties of the air have been assigned to the air domain surrounding the coil and the conductor block.

A hollow cylindrical conductor is defined with the multi-turn coil domain feature of Comsol. This enables one to specify coil's properties such as the number of turns, as well as the conductivity and the gauge of the wire. The multi-turn coil domain feature of Comsol is useful when modelling the inductors with a highly compact number of coil turns at low operating frequencies. In this case, it is possible to assume the current density is distributed uniformly over the coil’s cross-section. The coil is excited by an alternating current *I* with the magnitude of 1 A, and the current density of a circular multi-turn coil domain is calculated through

1 $$ {\varvec{J}}_{e} = NI/S $$

where *N* is the number of windings, and *S* is the cross-sectional area of the cylindrical object to which the coil domain has been assigned to.

It is worthwhile to mention that, according to Burke’s study, the scan direction is parallel to the notch’s length. So, to simulate this scan, the absolute coil is moved over a 20 mm range along the notch’s centerline. The corresponding coil impedance is computed for each displacement step of 2.5 mm starting at the center of the notch.

### Governing Electromagnetic Equations

The “Magnetic Field Physics” package in Comsol is selected for FE simulations. The governing equation for the magnetic field interface is based on the time-harmonic Maxwell-Ampere’s law [31].

2 $$ (\nabla \times (\nabla \times {\varvec{A}}))/\mu + (j\sigma \omega - \omega^{{2}} \varepsilon ){\varvec{A}} = {\varvec{J}}_{e} ,\;j = \sqrt { - 1} $$

where *µ* is the magnetic permeability, *σ* stands for electrical conductivity, ***A*** is the magnetic vector potential, *ε* is the electrical permittivity, *ω* is the angular frequency and ***J***_***e***_ is externally applied current density. Comsol solves Eq. (2) in the frequency domain.

In the low-frequency regime, where the electromagnetic wavelength is much greater than the size of the system, it is possible to assume that the quasi-static form of Maxwell’s equations can be used ($\sigma > > \omega \,\varepsilon$). Although in such a case, the displacement current term $\left( {\partial {\varvec{D}}/\partial t} \right)$ is generally excluded from the calculations, the equation embedded in the magnetic field package of Comsol takes it into account without introducing any additional computational cost.

### The Extent of the Simulation Domain

**Fig. 4:**
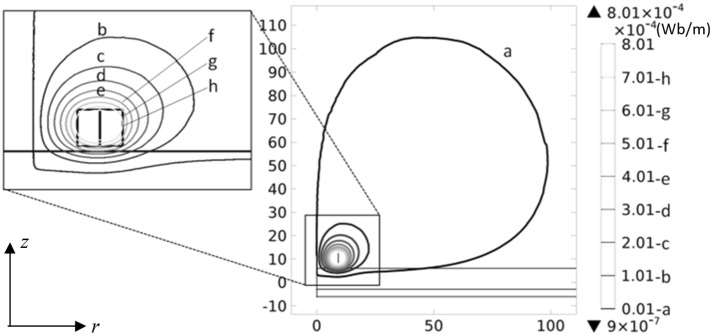
Contour of the φ component of the magnetic vector potential ***A*** given by a 2-D-axisymmetric model of an absolute coil located over an extremely large (500 mm × 500 mm × 12 mm) un-defective sample. r and z values are in mm. According to [28], simulation is performed with an operating frequency of 900 Hz

Generating an unbounded or infinitely extended air domain in FE simulations has been a long-standing problem for many physics packets. It is beneficial, in terms of resources and execution time, to truncate such a domain to an extent in which the simulation results remain reliable. Fortunately, in eddy current problems, the region of practical interest can be limited to a bounded domain large enough to capture a reasonable amount of the coil’s electromagnetic field. Air domain truncation should be done carefully, so to prevent the edge effects spoil the final results. To this end, the fields are initially solved according to a 2-D-axisymmetric model for a large 500 mm × 500 mm × 12 mm un-defective block surrounded by a 600 mm × 600 mm × 600 mm air domain. Figure 4 shows the contour plots of the *φ* component of the magnetic vector potential ***A*** projected on the *r*-*z* plane passing through the center of the coil, where *φ*, *r* and *z* are the components of a cylindrical coordinate system. The air domain is then truncated around the region where the intensity of the magnetic vector potential drops to 0.1% of its maximum value. The width and length of the conductive block domain are also shortened with respect to the truncated size of the air domain such that each of the block’s lateral faces is 10 mm apart from the nearest wall of the air domain. A new simulation is performed with this truncated model and the results do not reveal any changes in the field distribution. This shows no edge effects are introduced by truncating the model’s domains. It shall be noted that the changes in the distribution of the electromagnetic fields caused by the presence of a notch just occur locally around the notch itself. Hence, it is safe to assume that the field distribution at the boundaries of the air domain remains unchanged after introducing the notch. The truncated domain can, therefore, be used for further simulations of this absolute coil model.

### Mesh Assignment

A sound understanding of the impact caused by each FE parameter on the model is critical for attaining accurate simulation results. The mesh sizes (i.e. the density of finite elements) as well as the distribution of these elements within the model are certainly two of them. Mesh is a geometrical dependent feature. One should consider the physics used and the dimensions of the domain while generating the elements within each domain. It is also important to have a satisfactory resolution for the solved fields, which is fully dictated by the element’s type and size.

In the current problem, the aluminum block is partitioned using a cylindrical surface in order to create a more concentrated mesh underneath the coil. The diameter of the partitioned region is twice the diameter of the coil. This cylindrical region in the aluminum block moves together with the coil at each displacement step so as to always maintain the concentrated mesh underneath the coil.

Because of the skin effect, eddy currents are almost completely contained within the first three standard penetration depth (*δ*) as defined by

3 $$ \delta = \sqrt {\frac{2}{\omega \mu \sigma }} $$

Therefore, resolving the induced currents within their first three standard penetration depth is crucial for obtaining the correct values of the coil’s complex impedance. The standard penetration depth for the aluminum block is equal to 3.04 mm, as reported by Burke [28]. Initially a boundary mesh composed of 6 equal layers of second-order tetrahedral elements has been generated for this region within the aluminum block. Later, the number of layers has been optimized according to the sensitivity of the simulated signals to this parameter. Apart from these critical regions, free tetrahedral meshes have been created in the rest of the model’s geometry including the coil domain.

In order to get accurate simulation results in the shortest computational time, a sensitivity study is performed with respect to mesh size. For this study, the mesh is generated manually for each simulation step in order to have better control over its distribution within different regions. The sensitivity of the simulation results in terms of mesh sizes is studied in two stages. First, the number of boundary layer meshes is kept at a constant value (six across the 3*δ* depth within the aluminum block) while the maximum element to the coil thickness (6 mm) ratio is changed (i.e., the element’s size in the coil and aluminum block domains is refined in 3 steps). The computed impedance data are then compared to Burke’s measurements [28]. In each step, regardless of the total number of elements, the mesh sizes in the coil and aluminum block domains are equally scaled such that the corresponding maximum size ratio remains constant. This approach enables to keep the mesh quality within an acceptable level and avoid the adverse influence of low element quality in the solution. In the second stage of this study, the best maximum element size to the coil’s thickness ratio (S/T) obtained previously is applied to all domains and kept constant. The purpose of introducing the ratio is to relate the element size to the probe’s physical geometry since the two different absolute and differential configurations are investigated in the study, where the coil geometry can vary significantly from one configuration to another. Then, the number of boundary layer meshes is varied from 1 to 3 per *δ* depth within the aluminum block. It is noteworthy that for mesh sensitivity studies the conductivity of air is set to 1 S/m.

**Fig. 5:**
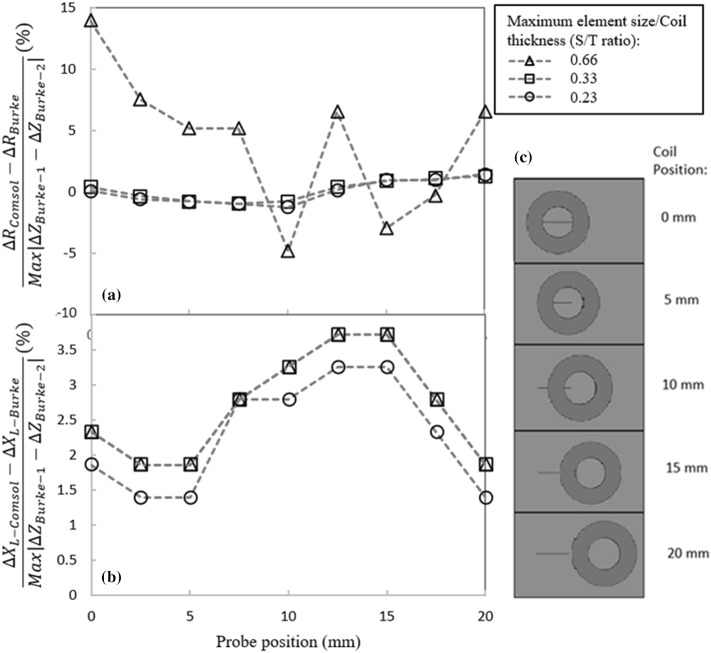
Effect of maximum element size to coil’s thickness ratio (S/T) on the normalized computed absolute coil’s **a** resistance and **b** inductive reactance as it scans an aluminum block containing a notch, **c** schematic of the coil position with respect to the notch location. Results are normalized by the magnitude of impedance variation in Burke’s measurements [28]

Figure 5a and b show the variations of the coil’s resistance and inductive reactance for different S/T values as the coil scans the aluminum block containing a notch, respectively. Resistance and inductive reactance values are normalized by the impedance measurements of Burke in these figures [28]. In the normalization process, at each scanning position, the coil’s inductive reactance and resistance measured by Burke are subtracted from the estimated values by simulations, and the results are divided by the maximum variations of the reported impedance measurements. Looking at the resistive part of impedance presented in Fig. 5a, $\Delta R_{Comsol}$ is associated with the calculated probe’s resistance at each position. This value is subtracted by the measured resistance ($\Delta R_{Burke} )$ at the same scan position. Finally, the subtraction results for all scan positions were divided by the maximum impedance value measured by Burke (i.e. $\left| {\Delta Z_{Burke\_1} - \Delta Z_{Burke\_2} } \right|$). The point of this normalization scheme is to subtract the measured impedance values, provided by Burke, from our computed impedance values, and report the difference (i.e. error in simulation) in percentage. This normalization strategy also allows examining the convergence of the solver as different S/T ratios are selected. For better understanding, the coil scanning positions relative to the notch are shown schematically for 5 different coil displacements in Fig. 5c. According to Fig. 5a, changing the S/T ratio from 0.23 to 0.33 does not introduce any significant change in the resistive part of the impedance and, regardless of the scan position, the error always remains below 2%. However, the solver becomes unstable for an S/T ratio of 0.66 resulting in unreliable resistance values for the coil. This means that S/T ratio needs to be small enough to capture the resistive losses caused by eddy currents inside the conductor. On the other hand, the error connected to the coil’s inductive reactance has the same values for S/T ratios of 0.33 and 0.66 position wise. Furthermore, as the S/T ratio is further reduced to 0.23, the inductive error is decreased by only 0.5%. Although impedance estimations with the lowest level of error are achieved through meshing the block with the smallest S/T of 0.23, such a parameter increases the computational expenses unreasonably. Therefore, to avoid lengthy computational time, a S/T ratio of 0.33 is selected knowing that the solver converges to consistent impedance estimations.

**Fig. 6:**
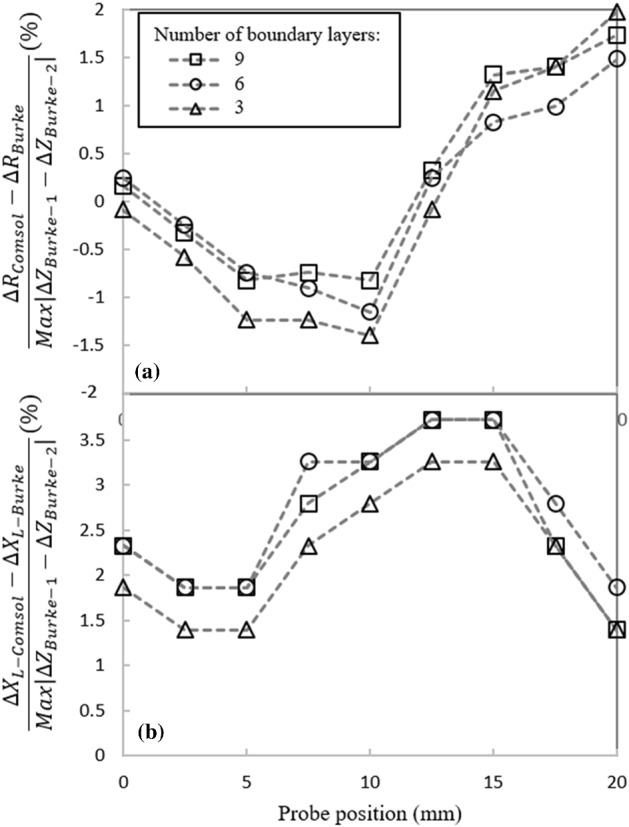
Effect of the number of boundary layers applied in the first three eddy current penetration depths on the normalized computed absolute coil’s **a** resistance and **b** inductive reactance as the coil scans an aluminum block containing a notch. Results are normalized by the magnitude of impedance variation in Burke’s measurements [28]

Figure 6a and b depict the variations of the normalized coil’s resistance and inductive reactance as a function of the coil’s position for three different numbers of boundary layer meshes. The normalization scheme is similar to the one used in the previous case. As it can be observed in Fig. 6a, the coil’s resistance is underestimated up to the scan position of 10 mm. This error is larger for 3 boundary layers as compared to the one obtained by 6 and 9 boundary layers. The estimation error becomes positive for the remaining probe positions where 6 boundary element layers provide the best estimation. In Fig. 6b, computed inductive reactance values are overestimated at all scan positions, which is similar to the behavior seen in the previous sensitivity study. It is evident from the figure that using 6 and 9 boundary layers in the model result in very close computed values at each scan position. This suggests the solver already converges with 6 boundary layers and that applying additional boundary layers would undoubtedly result in an undesired increase of the computational time. Accordingly, 6 boundary layered elements should be sufficient across the first 3*δ* depth. The total number of elements used in the model can vary between 400,000 to 800,000, approximately, depending on the number of boundary layers defined in the sample. Solving such a model for one scanning position in a direct frequency-domain study of Comsol, takes from 15 to 40 min when using a desktop personal computer (PC) configured with an Intel© Core i7 processor with base frequency of 2.60 GHz, and a double data rate 3 (DDR3)—32 Gigabytes (GB) of random access memory (RAM).

### Computed Impedance with the Optimized Model Parameters

**Fig. 7:**
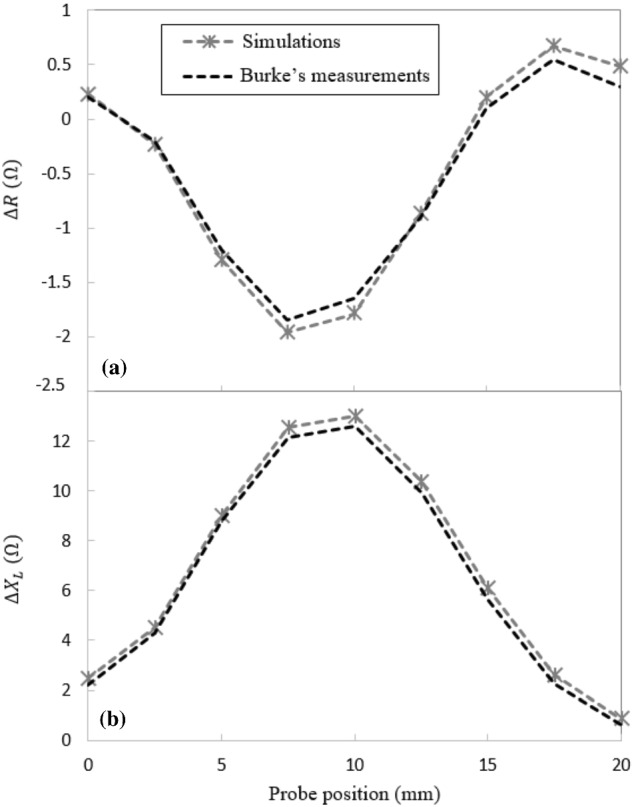
Calculated values of the absolute coil’s **a** resistance and, **b** inductive reactance as the coil scans over a notch. Comparison with impedance measurements extracted from Burke's work [28]

The optimized simulation parameters are applied to the model, as concluded from the previous sensitivity studies. Subsequently, the estimated values for the resistive and the inductive parts of the impedance are superimposed on Burke’s measurements in Fig. 7a and b, respectively. Although Fig. 7 shows a quite good fit with experimental data, a small discrepancy between the calculated and the measured impedance components is observed at each scan position. The computed inductive reactance is always at least 1.5% higher than the measured values, regardless of the parameters in use. A deeper investigation into the model indicates that the reference edge specified within the circular coil’s model, which determines the direction of current flow in a relatively thick-sectioned coil, can affect the computed coil’s impedance. Based on the instructions provided by Comsol for modelling 3-D coils [32], choosing a closed-loop running through the middle of the circular coil’s thickness is expected to provide the best-computed impedance values. In an attempt to improve the accuracy of the model, the effect of the position of the reference edge would thus require further investigation. Fortunately, this issue is not a concern in the modelling of split-D probes, where the coils’ cross-sections are far thinner (i.e., one or two layers of AWG 42 wires). Additionally, they are modelled through numeric multi-turn coil domains instead of circular multi-turn coil domains. In such a modelling tool, there is no need to specify reference edges since the direction of the current is determined by defining an additional step within the solver.

## FEM Analysis of a Split-D Differential Coil

A half 3-D CAD model for a differential probe is developed in Comsol based on the probe’s dimensions presented in Table 1. Magnetic insulation boundary condition within AC/DC module is assigned to the plane of symmetry of the half model in order to effectively consider the missing half of the problem in modelling. Instead of generating a complex CAD model for the coils including all coil turns and the corresponding geometrical complications such as winding angles and separations, three solid domains are generated for the coils, and the multi-turn coil domain within Comsol is assigned to each of them. Two solid geometries are created for the cores by cutting a cylinder in half across its diameter. A hollow cylinder is also created for the probe’s shielding, and all the components are assembled properly respecting the dimensions of the commercial split-D probe. A very small 0.03 mm lift-off is introduced to the probe’s model according to the experimental setup.

Unlike the scan path used in the experimental tests (− 2 mm to + 2 mm), it is optimized for simulations to shorten the time of the solver. Therefore, simulations are performed in the range of 0 to 1.3 mm (i.e., starting from a position where the center of the coil is aligned with the notch’s centerline, and ending at a position where the coil is 1.3 mm away from the notch’s centerline). The complete impedance signal is supposed to be a perfect mirror image of the signal so obtained, resulting in the distinctive 8-shaped signal. Usually, the simulations at 0.1 mm steps along the scanning path are carried out altogether in a single run. In a few cases, however, due to the complexity of the mesh structure, each 0.1 mm scanning step is treated individually.

**Fig. 8:**
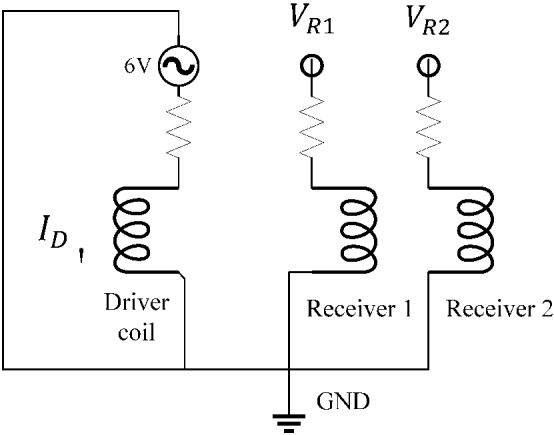
A schematic of the probe’s circuit consisting of an alternating voltage source that is used for exciting a driver coil, and the two receiver coils in which a voltage is induced by the driver coil. The differential voltage across these receiver coils is measured during the scan

A 6 V, 500 kHz alternating voltage is applied to the driver coil and both receiver coils are considered open (no current flow through them) as demonstrated in Fig. 8. At every scanning step, the voltage across each of the two receiver coils is obtained (${\text{V}}_{R1}$ and ${\text{V}}_{R2}$). As given by Eq. 4, the differential impedance of the probe $\left( {\Delta {\varvec{Z}}} \right)$ can be expressed as the difference of voltages ${\text{V}}_{R1}$ and ${\text{V}}_{R2}$ divided by the current flowing in the driver coil $\left( {{\text{I}}_{D} } \right)$.

4 $$ \Delta {\varvec{Z}} = ({\text{V}}_{R2} - {\text{V}}_{R1} )/{\text{I}}_{D} $$

$\Delta {\varvec{Z}}$ represents the variation of the probe impedance with respect to the one obtained in sound areas of the sample since the differential voltage for an un-defective block is extremely small (theoretically zero).

The Nortec-500 flaw detector, the equipment used to experimentally acquire impedance data in the current work, provides relative impedance values rather than absolute ones. Therefore, in order to enable a comparison between simulated and measured impedances, data conversion procedure is required. As previously mentioned, the relative probe impedance, which is displayed on the Nortec screen as a moving dot, is collected through the acquisition of two voltage signals (***V***_***H***_ and ***V***_***V***_), which are respectively proportional to the horizontal and the vertical positions of the dot on the screen. These two voltages are first subtracted by the horizontal and vertical components of the nulled impedance locus (*i.e. V*_***H0***_ and ***V***_***V0***_), which normally falls on the origin of the impedance plane, and then combined into a complex phasor given by.

5 $$ \Delta {\varvec{V}}_{Nortec} = \left( {{\varvec{V}}_{H} - {\varvec{V}}_{H0} } \right) + \left( {{\varvec{V}}_{V} - {\varvec{V}}_{V0} } \right) \cdot j = M\angle \theta $$

6 $$ M = \sqrt {\left( {{\varvec{V}}_{H} - {\varvec{V}}_{H0} } \right)^{2} + \left( {{\varvec{V}}_{V} - {\varvec{V}}_{V0} } \right)^{2} } $$

and

7 $$ \theta = \tan^{ - 1} \left( {\frac{{{\varvec{V}}_{V} - {\varvec{V}}_{V0} }}{{{\varvec{V}}_{H} - {\varvec{V}}_{H0} }}} \right) $$

Comparison between measured $\Delta {\varvec{V}}_{Nortec}$ and simulated $\Delta {\varvec{Z}}$ shows that with the horizontal and vertical gains set to 56 dB on Nortec system, $\Delta {\varvec{V}}_{Nortec}$ shall be systematically multiplied by 0.0016 and phase-shifted by 22 degrees clockwise to get a gain compensated $\Delta {\varvec{V}}_{2}$, which is the value that can be compared to the corresponding computed impedance ($\Delta {\varvec{Z}}$). The value 0.0016 is deduced from the following relationship

8 $$ Gain(dB) = 20\log \left(\frac{{\Delta {\varvec{V}}_{Nortec} }}{{\Delta {\varvec{V}}_{2} }}\right) $$

This phase shift is also believed to be due to the Nortec amplification circuitry that could introduce a gain and a frequency-dependent phase shift in the displayed signal.

Following the same development described in Sect. 3 for the modelling of the absolute coil, a sensitivity analysis is carried out for the case of the split-D differential probe to understand how the changes in the model’s parameters affect the simulation results.

### The Extent of the Simulation Domain

**Fig. 9:**
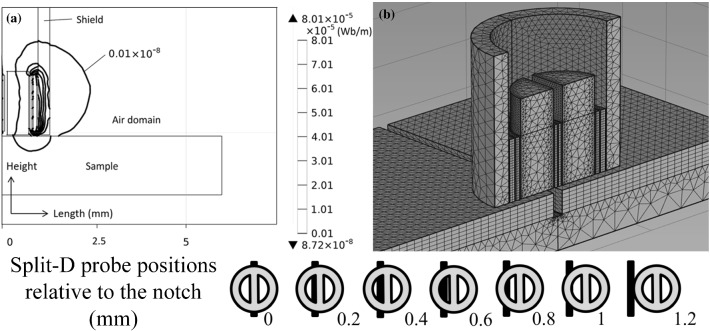
**a** Contour map of the magnetic vector potential component perpendicular to the symmetry plane at 500 kHz for a split-D probe over an un-defective aluminum block. **b** Model’s mesh distribution for a split-D probe scanning an aluminum block with a 0.503 mm deep notch

The effective probe footprint may vary depending on the probe lift-off, the operating frequency, the probe shielding, the coils' geometry, and their configuration. Accordingly, the truncation sizes of the air and aluminum block domains, as deduced in Sect. 3, are no longer applicable to the case of the split-D probe since the operating frequency is significantly higher and the probe geometry much smaller. However, the air and aluminum block domains are still truncated following the same strategy based on the magnitude of the magnetic vector potential in order to reduce the computation time. Figure 9 illustrates the contour map of the magnetic field potential component perpendicular to the symmetry plane at 500 kHz along with the model’s initial mesh distribution. The outermost contour shows the region at which the amplitude of vector potential reduces to approximately one-thousandth of its maximum value. Consequently, the air domain can be truncated up to twice the size of the shielding diameter as the vector potential field is concentrated inside the shielding.

### Mesh Size

Following the methodology used in Sect. 3.3, the maximum element size in the aluminum block domain to driver coil thickness ratio (i.e. S/T ratio) is changed in 4 steps while the element size of other domains is scaled proportionally. The thickness of the driver coil for the split-D probe is 0.32 mm considering the wire diameter and its stacking order in the coil. Worth to mention that an air conductivity value of 1 S/m is used for these simulations and the standard penetration depth in aluminum block is 0.16 S/m.

**Fig. 10:**
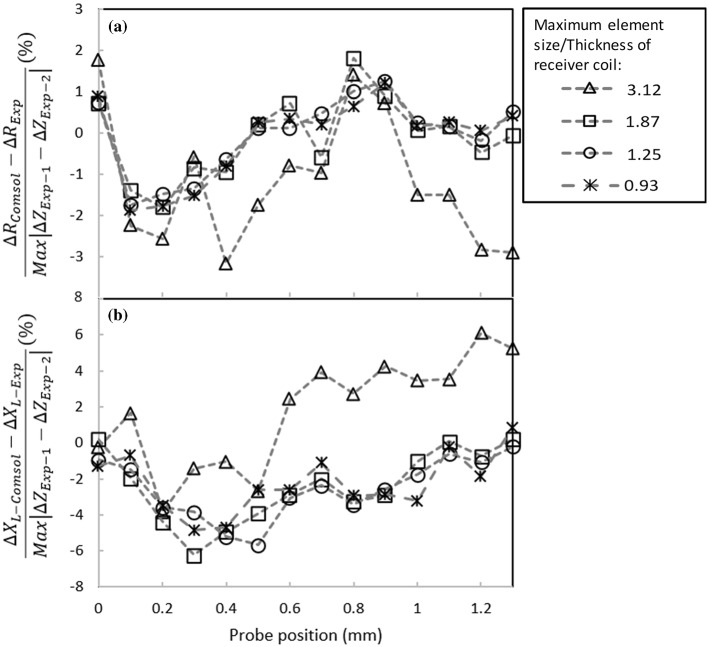
Effect of the element size on the normalized computed **a** real and **b** imaginary parts of the differential impedance for a 0.188 mm deep notch in an aluminum block. Simulations are performed using four different numbers of volumetric tetrahedral elements

**Fig. 11:**
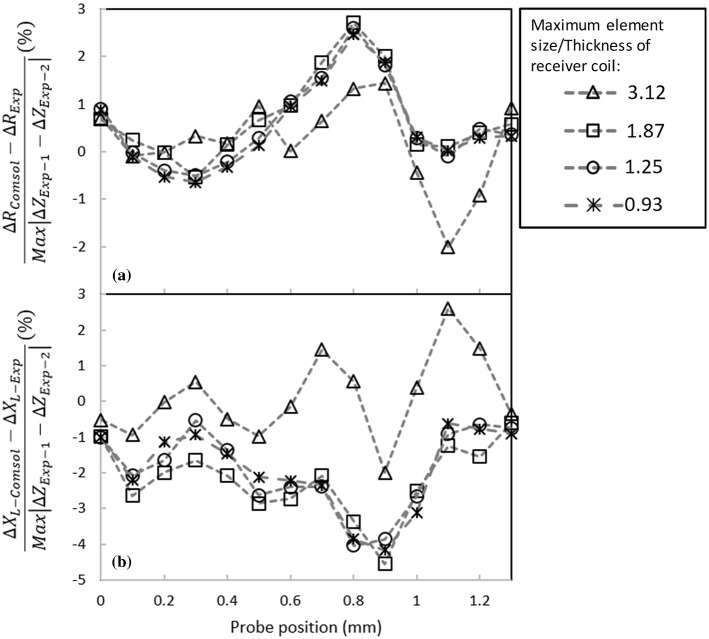
Effect of the element size on the normalized computed **a** real and **b** imaginary parts of the differential impedance for 1.008 mm deep notch in an aluminum block. Numerical simulations are performed using four different numbers of volumetric tetrahedral elements

Figures 10 and 11 show the variations of the real and imaginary parts of the normalized probe’s impedance scanning over the 0.188 mm and the 1.008 mm deep notches, respectively. These figures are plotted for 4 different S/T values. The same normalization scheme used in Sect. 3 is applied here however, the curves are normalized by the impedance measurements of the notches using the split-D probe. The figures show that imaginary and real parts of the computed impedance converge as the ratio of 1.87 is used in the model. In order to leave some room for higher resolution results, the ratio of 1.25 will be used for this probe model in the following simulations. Given the ratio selected here, the model consists of approximately 900,000 s order elements. It takes the solver about 45 min to process each scan step of the simulation using the PC configurations mentioned earlier.

### Simulation Results and Discussion

**Fig. 12:**
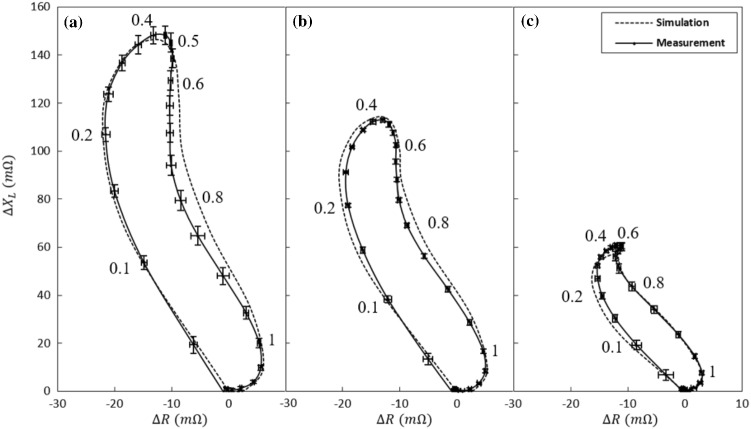
A single loop of the 8-shaped signals obtained by plotting the imaginary and real parts of the probe’s impedance on the impedance plane for **a** 1.008 mm, **b** 0.503 mm, and **c** 0.188 mm deep notches

The response of the split-D probe is here investigated numerically and experimentally as it scans three EDM notches of different depths in an aluminum block. Imaginary and real parts of the differential probe impedance are measured and numerically calculated. These values are plotted together, on an impedance plane, in Fig. 12 for 1.008 mm, 0.503 mm, and 0.188 mm deep notches. All the three impedance planes of Fig. 12 are shown with the same scale to ease comparison between the given signals. Again, only a single loop of the 8-shaped signal is shown because of the symmetry of the model.

**Fig. 13:**
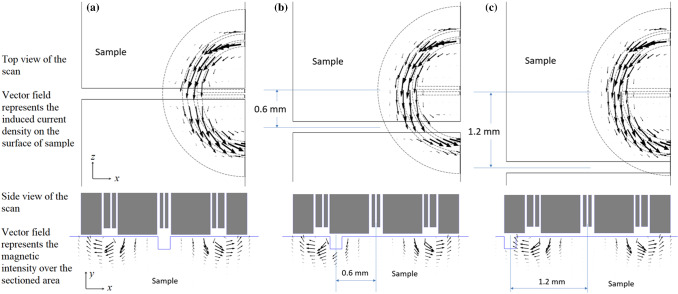
Distribution of the *z* and *x* components of the induced current density in the vicinity of the 0.188 mm deep notch as the probe is displaced by **a** 0 mm, **b** 0.6 mm and **c** 1.2 mm. Side view of the scan shows the distribution of the *x* and *y* components of the magnetic field intensity

Referring to Figs. 13 it is observed that the shape of simulated and measured signals is in good agreement for all the notches. The most significant discrepancy appears in the imaginary component of the impedance. From Fig. 10 (the shallowest notch), it could be seen that the imaginary part is underestimated by 6% at a probe position of about 0.5 mm. This discrepancy in the imaginary part of the probe impedance is reflected in the signal peak of the simulated complex impedance loops in Fig. 12c. Moreover, referring to Fig. 11 (the deepest notch) the signal is underestimated by 4% at the scan position of 0.8 mm. This deviation of simulation form measurement appears as a widening of the simulated signal in Fig. 12a. Otherwise, most of the simulated impedance values are contained within the measurement error. To derive the error, each of these notches is scanned 5 times using the split-D probe and the impedance variations in each scan are recorded. Afterward, the mean value and the standard deviation ($\sigma$) of the 5 impedance measurements are calculated for each notch. Accordingly, the measurement error is presented by $\pm \sigma$ at each probe’s position.

Discrepancies between simulated and measured impedances are believed to be related to the deviation of the geometry of the manufactured notches from the ideal simulated ones (rectangular slot). In an EDM process, thin electrodes are used to erode narrow surface notches. As the notch gets deeper, the electrode’s lateral faces may further remove metal from the notch walls. Consequently, the resulting notch is wider in the vicinity of its opening than at its tip. This nonuniformity grows by increasing the nominal depth of a notch. Correspondingly, referring to Fig. 12, we find that the largest discrepancy in the width of the complex impedance loops is occurring with the deepest notch. Dimensions and properties of the probe components used in the model could also contribute to the observed discrepancies. In addition, the sensitivity of the probe’s signal to geometrical imperfections grows significantly as the notch gets shallower. Hence, the largest discrepancy between simulations and measurements is observed for the shallowest notch.

The desired precision level for each feature of a signal, such as amplitude, phase, and shape, is strongly application oriented. For instance, in order to have a good estimation of the crack characteristics in inversion approaches, the crack signals shall be accurately reproduced using modelling. However, the importance of signal shape and phase is undoubtedly less in reliability studies (POD studies) where the signal amplitude is the primary influential factor [33, 34]. Nonetheless, results reported in this work confirm that Comsol model can reasonably predict probe impedance variations (in shape and amplitude) as it is scanned across a notch.

The use of such a model could also help to properly design a specific probe as the impact of design parameters on probe performances could be accurately simulated. Indeed, in addition to probe impedance, magnetic and electric vector fields could be displayed to understand the probe behavior. As an example of this, Fig. 13 shows the surface distribution, using the vector field representation, for the *z* and *x* components of ${\varvec{J}}_{in}$ (induced current density) in the vicinity of 0.188 mm deep notch for three different probe positions over this notch. Moreover, the surface distribution for the *y* and *x* components of $H$(magnetic field intensity) is depicted beneath these figures as well. As demonstrated, the surface current density is significantly perturbed at the scan positions of 0 mm and 0.6 mm. At the scan position of 0 mm, the perturbation is seen by both of the receiver coils equally. However, the notch and its corresponding perturbation zone are directly located underneath one of the receiver coils at the scan position of 0.6 mm. This results in the maximum amplitude of the differential impedance of the receiver coils for the shallowest notch.

## Summary and Conclusions

In this paper, a model-based study of an absolute probe and a split-D reflection differential surface probe is performed using Comsol Multiphysics. The half 3-D model of the absolute coil is generated, and the numerical simulations are carried out for the scan of a rectangular notch located on the surface of an aluminum block as per Burke’s benchmark problem [34]. In an attempt to obtain reliable results, sensitivity to model mesh size has been studied. Simulations revealed that the optimum mesh parameter is to use 6 boundary layer meshes across the first three penetration depths in the conductor block, with a maximum element size to coil’s thickness ratio (S/T) of 0.33. Based on these parameters, a quite good agreement is achieved between the numerical results and the benchmark measurements [34]. In fact, the error on the estimation of the coil's resistance is less than 2% of the impedance variation range. This error is found to be less than 4% for the coil's inductive part. It has also been noted that the coil's inductive reactance was always overestimated by the model (by at least 1.5%) most likely because of a parameter used to define the direction of current flow in a relatively thick-sectioned coil.

Taking advantage of the knowledge acquired from the Comsol simulation of the absolute probe, a half 3-D model of a commercial split-D reflection differential surface probe has been investigated as well. Similar to the previous case, a sensitivity study to the element size has been carried out. For this smaller probe operating at a much higher frequency (500 kHz), a S/T ratio of 1.87 has been found to be optimum. Again, 6 boundary layer meshes across the first three penetration depths in the conductor block have been used. Based on these parameters, the computed impedance values closely match the measurements. Only a slight widening of the simulated complex impedance loops has been observed. This minor discrepancy is believed to be due to non-ideal notch geometry in the measurements. The other factors contributing to this error could be inaccuracies in dimensioning of the probe constituents and or in properties of the numerical probe model.

The present work aimed to explore the potential of Comsol Multiphysics to accurately estimate a split-D probe impedance while it scans surface cracks. Results obtained herein seem to confirm that signal amplitude could be simulated to a level of accuracy that should be sufficient for the probability of detection studies. However, if the goal is to characterize the crack depth and shape through a fuzzy logic inversion algorithm, accuracy of the model outputs shall be further improved. To do so, information about probe’s material and geometrical parameters needs to be better defined. This aim will be pursued in future investigations.

## References

[1] COMSOL I: COMSOL Multiphysics Reference Manual, version 5.3. COMSOL AB. (2018) www.comsol.com. Accessed 1 Mar 2018

[2] Jones R, Molent L, Pitt S (1999). Study of multi-site damage of fuselage lap joints. Theor. Appl. Fract. Mech..

[3] Pitt S, Jones R (1997). Multiple-site and widespread fatigue damage in aging aircraft. Eng. Fail Anal..

[4] Bhaumik S, Sujata M, Venkataswamy M (2008). Fatigue failure of aircraft components. Eng. Fail Anal..

[5] Findlay S, Harrison N (2002). Why aircraft fail. Mater. Today.

[6] Grover HJ (1966). Fatigue of Aircraft Structures.

[7] Davis JR (1989). ASM Handbook: Nondestructive Evaluation and Quality Control.

[8] Hellier C (2001). Handbook of Nondestructive Evaluation.

[9] Shull PJ (2002). Nondestructive Evaluation: Theory, Techniques, and Applications.

[10] García-Martín J, Gómez-Gil J, Vázquez-Sánchez E (2011). Non-destructive techniques based on eddy current testing. Sensors.

[11] Krause, H., Hohmann, R., Gruneklee, M., Maus, M.: Aircraft wheel and fuselage testing with eddy current and SQUID. In: 7th European Conference on Non-destructive Testing, vol. 42, No. 3, pp. 148–151 (2000)

[12] Wincheski B, Namkung M (1998). Detection of sublayer fatigue cracks under airframe rivets. Review of Progress in Quantitative Nondestructive Evaluation.

[13] Hagemaier D, Kark G (1997). Eddy current detection of short cracks under installed fasteners. Mater. Eval..

[14] Khan, T., Nakagawa, N.: Quantitative impedance measurements for eddy current model validation. Review of Progress in Quantitative Nondestructive Evaluation: Volume 19, vol. 509, No. 1, pp. 441–448 (2000). 10.1063/1.1306082

[15] Mooers, R., Knopp, J., Blodgett, M.: Model based studies of the split D differential eddy current probe. In: Review of Progress in Quantitative Nondestructive Evaluation: Volume 31, vol. 1430, No. 1, pp. 373–380 (2012). 10.1063/1.4716252

[16] Mooers, R.D., Knopp, J.S., Aldrin, J.C., Sathish, S.: Split D differential probe model validation using an impedance analyzer. In: 40th Annual Review of Progress in Quantitative Nondestructive Evaluation: Incorporating the 10th International Conference on Barkhausen Noise and Micromagnetic Testing, vol. 1581, No. 1, pp. 1511–1518 (2014). 10.1063/1.4865002

[17] Mooers, R.D., Knopp, J.S., Aldrin, J.C., Sathish, S.: Simulated parametric study based on a representative split D differential eddy current probe. In: 40th Annual Review of Progress in Quantitative Nondestructive Evaluation: Incorporating the 10th International Conference on Barkhausen Noise and Micromagnetic Testing, vol. 1581, No. 1, pp. 1344–1351 (2014). 10.1063/1.4864977

[18] Nakagawa, N., Khan, T., Gray, J.: Eddy current probe characterization for model input and validation. In: Review of Progress in Quantitative Nondestructive Evaluation: Volume 19, vol. 509, No. 1, pp. 473–480 (2000). 10.1063/1.1306086

[19] Nakagawa, N., Yang, M., Larson, B.F., Madison, E., Raulerson, D.: Study of the effects of EDM notch width on eddy current signal response. In: 35th Annual Review of Progress in Quantitative Nondestructive Evaluation, vol. 1096, No. 1, pp. 287–294 (2009). 10.1063/1.3114217

[20] Rosell, A., Persson, G.: Modelling of a Differential Sensor in Eddy Current Non-destructive Evaluation. COMSOL Conference (2011)

[21] Sabbagh, H.A., Sabbagh, E.H., Murphy, R.K.: (2002) Recent advances in modeling eddy-current probes. In: Review of Progress in Quantitative Nondestructive Evaluation, vol. 615, No. 1, pp. 423–429 (2002). 10.1063/1.1472829

[22] Aldrin, J.C., Sabbagh, H.A., Murphy, R.K., Sabbagh, E.H., Knopp, J.S., Lindgren, E.A., Cherry, M.R.: Demonstration of model-assisted probability of detection evaluation methodology for eddy current nondestructive evaluation. In: Review of Progress in Quantitative Nondestructive Evaluation, vol 1. AIP Publishing, pp. 1733–1740 (2012). 10.1063/1.4716421

[23] Shell, E.B., Aldrin, J.C., Sabbagh, H.A., Sabbagh, E., Murphy, R.K., Mazdiyasni, S., Lindgren, E.A.: Demonstration of model-based inversion of electromagnetic signals for crack characterization. In: 41st Annual Review of Progress in Quantitative Nondestructive Evaluation: Volume 34, vol. 1650, pp. 484–493 (2015). 10.1063/1.4914645

[24] Knopp, J.S., Aldrin, J., Lindgren, E., Annis, C.: Investigation of a model‐assisted approach to probability of detection evaluation. In: Review of Progress in Quantitative Nondestructive Evaluation, vol. 894, No. 1, pp. 1775–1782 (2007). 10.1063/1.2718178

[25] Aldrin, J., Knopp, J., Lindgren, E., Jata, K.: Model-assisted probability of detection (MAPOD) evaluation for eddy current inspection of fastener sites. In: Review of Progress in Quantitative Nondestructive Evaluation, vol. 28, pp. 1784–1791 (2009)

[26] Knopp JS, Aldrin JC, Misra P (2006). Considerations in the validation and application of models for eddy current inspection of cracks around fastener holes. J. Nondestruct. Eval..

[27] Verite, J. (1990) A coil over a crack (results for benchmark problem 8 of team workshop). In: COMPEL-The International Journal for Computation and Mathematics in Electrical and Electronic Engineering, vol. 9, No. 3, pp. 155–167. 10.1108/eb010072

[28] Burke S (1988). A benchmark problem for computation of δz in eddy-current nondestructive evaluation (NDE). J. Nondestruct. Eval..

[29] Xu, B., Xie, W., Viens, M., Mohseni, E., Birglen, L., Mantegh, I.: Intelligent eddy current crack detection system design based on neuro-fuzzy logic. In: International Workshop on Smart Materials and Structures, NDT in Canada 2013 Conference & NDT for the Energy (2013)

[30] Mooers, R.D., Aldrin, J.C.: Effects of angular variation on split D differential eddy current probe response. In: AIP Conference Proceedings, vol. 1. AIP Publishing, p. 090022 (2016)

[31] COMSOL I: AC/DC Module User’s Guide. COMSOL AB (2007)

[32] COMSOL I: Single-Turn and Multi-Turn Coil Domains in 3D. COMSOL AB (2012)

[33] Underhill P, Krause T (2011). Enhancing probability of detection and analysis of bolt hole eddy current. J. Nondestruct. Eval..

[34] Lemire H, Underhill P, Krause T, Bunn M, Butcher D (2010). Improving probability of detection of bolt hole eddy current inspection. Res. Nondestr. Eval..

